# Approach to COVID-19 in older adults and indications for improving the outcomes

**DOI:** 10.1080/07853890.2023.2265298

**Published:** 2023-10-15

**Authors:** Claudio Tana, Livia Moffa, Katia Falasca, Jacopo Vecchiet, Marco Tana, Cesare Mantini, Fabrizio Ricci, Andrea Ticinesi, Tiziana Meschi, Francesco Cipollone, Maria Adele Giamberardino

**Affiliations:** a Geriatrics Clinic, SS Annunziata Hospital of Chieti, Chieti, Italy; b Infectious Disease Department and COVID-19 Unit, University Hospital of Chieti, Chieti, Italy; c Internal Medicine Unit, SS. Annunziata Hospital of Chieti, Chieti, Italy; d Department of Neuroscience, Imaging and Clinical Sciences, "G. D’Annunzio" University of Chieti-Pescara, Chieti, Italy; e Department of Medicine and Surgery, University of Parma, Parma, Italy; f Italy and Geriatric-Rehabilitation Department, Azienda Ospedaliero-Universitaria di Parma, Parma, Italy; g Medical Clinic, SS. Annunziata Hospital of Chieti, Department of Medicine and Science of Aging, "G. D’Annunzio" University of Chieti-Pescara, Chieti, Italy; h Department of Medicine and Science of Aging, “G. D’Annunzio” University of Chieti, Chieti, Italy

**Keywords:** Covid-19, SARS-CoV-2, older, age, adults, frailty

## Abstract

**Background: **COVID-19 continues to present challenges in the care of older adults with frailty and/or comorbidities and very old patients, who can be hospitalized with severe COVID-19 despite full vaccination. Frailty is a heterogeneous syndrome characterized by an increased aging-related vulnerability due to a reduced physiological reserve and function of systemic organs, and is associated with an impairment of activities of daily living. Frail older adults remain at elevated risk of mortality from COVID-19 compared to older adults without frailty, and some pre-existing risk factors such as malnutrition, prolonged bed rest, and the association with comorbidities can aggravate the SARS-CoV-2 infection. Furthermore, the severity of COVID-19 can impact on long-term functioning of older patients surviving from the infection. Persistent symptoms are another emerging problem of the post-vaccination phase of pandemic, as most patients suffer from chronic symptoms which can become debilitating and affect the daily routine. **Aim of this review:** In this complex relationship, the evaluation of COVID-19 in vulnerable categories is still a matter of high interest and personalized care plans based on a comprehensive geriatric assessment, tailored interventions; specific therapeutic algorithms among older adults are thus recommended in order to improve the outcomes.

## Introduction

The COVID-19 pandemic, caused by the rapid diffusion of the novel coronavirus SARS-CoV-2, has rapidly spread in the world since December 2019. About seven million people died during the COVID-19 pandemic [1]. The World Health Organization (WHO) has estimated that millions of older people have died without even being counted. Although COVID-19 can affect people of any age, older adults have been the most affected by severe forms of the disease. Poor outcomes among this population are associated with comorbidities such as diabetes, hypertension, respiratory and cardiovascular diseases. Another important prognostic factor in the evolution of COVID-19 is frailty [2]. In this narrative overview, the main pathophysiological, epidemiological and clinical features of COVID-19 in older adults are summarized. Main intervention measures are provided in order to try to improve the outcome of COVID-19 older adults at high risk of complications and mortality.

### Frailty and epidemiology in older adults with COVID-19

Frailty is a heterogeneous syndrome and there is no consensus about its correct definition. Several risk factors of frailty have been identified, such as malnutrition, inactivity and polypharmacy and patients with frailty syndrome are at risk for poor clinical outcomes such as hospital admission, reduced mobility, high risk of falls, mortality and cumulative decline in multiple physiological systems [3,4].

WHO defines frailty as a condition characterized by an increased aged-related vulnerability due to a reduced physiological reserve and function of systemic organs, and is associated with an impairment of activities of daily living (ADL) [5–10].

Frailty is very common in adults older than 70 years, and the incidence of frailty increases progressively with age. It has indeed been estimated that about 15% and 25% of subjects older than 65 and 85 years are affected by frailty, respectively.

Frailty is a dynamic and potentially reversible process which affects more often older women than men, who are however more susceptible to deterioration [11–13].

In the context of COVID-19, frail older adults were approximately 51% of hospitalized patients with elevated risk of mortality compared to older adults without frailty. Since the beginning of the SARS-CoV-2 pandemic, older adults have been the most affected group by severe COVID-19. In March 2020, the Centre for Disease Control and Prevention (CDC) has reported that adults aged > 65 years accounted for 80% deaths from COVID-19 [14–16]. It has been hypothesized that some pre-exiting risk factors such as malnutrition, prolonged bed rest, and the association with comorbidities can aggravate the SARS-CoV-2 infection [17–19], and although frailty has not been associated with an increased risk of SARS- CoV-2 infection [20–21], frail older adults seem to be at a higher risk of severe COVID-19 disease than non-frail older adults. A prospective cohort study investigating the risk of severe disease within 60 days from hospital admission in 114 older patients (median age, 67 years, of which 50% men) with confirmed COVID-19 pneumonia, has demonstrated that 37.7% of patients developed severe disease including eight cases of death, and the risk of progression was highest in pre-frail and frail patients (respectively in 15 of 39 pre-frail −38.5%- and 24 of 36 frail −66.7%-), the risk remaining higher in patients with versus those without frailty also after adjustment for age, sex, body mass index and laboratory data (hazard ratio -HR- of 5.01, 95% CI 1.16–21.61, *p* = 0.03 and of 7.47, 95% CI 1.73–32.34, *p* = 0.007 for pre-frail and frail patients, respectively) [22].

The severity of COVID-19 seems to impact also on long-term functioning of older patients surviving from the infection. Persistent symptoms are another emerging problem of the post-vaccination phase of the pandemic, as most patients suffer from chronic symptoms which can become debilitating and affecting the daily routine. The presence of persistent symptoms after an acute infection from SARS-CoV-2 has been defined ‘long COVID’ [23,24]. Also age itself and comorbidities had an impact on functional decline, and a significant association has been found with age (OR = 1.08, *p* = 0.028), a history of depressive disorder (OR = 3.05, *p* = 0.016), stroke (OR = 4.57, *p* = 0.003) and complications (OR = 2.24, *p* = 0.039) at a multivariate analysis [25]. These data confirm the high impact of COVID-19 in older adults and the complex relationship between frailty and COVID-19 [25,26].

## Pathophysiology of COVID-19 in older adults

Some hypotheses have tried to explain the differences in terms of risk of morbidity and mortality of older adults with COVID-19, but few studies have investigated the mechanisms underlying the difference of risk between older and younger people. Potential mechanisms of explanation are immunosenescence, microbial dysbiosis and inflamm-ageing [27].

SARS-CoV-2 is transmitted through respiratory droplets from person to person. The virus enters in host cells by binding angiotensin-converting enzyme 2 (ACE-2) receptors and can spread to the lungs by infecting pneumocytes, also called alveolar type 2 epithelial cells. They are not the only cells infected because another molecule, the lymphocyte function-associated antigen 1 (LFA-1), mediates the entry of SARS-CoV-2 into T lymphocytes [28].

Some people, especially older adults, are at high risk of acute respiratory distress syndrome (ARDS), because they have a significant reduction of ACE2 activity related to aging which leads to the increase of severity of lung injuries [29]. Furthermore, older adults have a decline in the numbers of cilia in the airway, which is associated with a reduced clearance of SARS-CoV-2 virus. It has been demonstrated, also before the pandemic, that older adults have an altered crosstalk between innate and adaptive immunity for an adeguate response against pathogens [30]. In normal conditions, antigen-presenting cells (APCs), *via* Toll-like receptors (TLRs), recognize pathogen-associated molecular patterns (PAMPs). This mechanism is impaired in older adults and contributes to the significant increase of injury in COVID-19 patients.

### Immunosenescence and inflamm-ageing

Immunosenescence is an impairment of the immune system associated with ageing. In older adults the immune system has a decreased capacity to cope with infections, which hampers the pathogen clearance.

The other immune mechanism which occurs with aging is inflamm-ageing, a chronic increase of systemic inflammation. The immune system is always hyperactive, but in an ineffective way [31].

Inflamm-aging leads to an increased production of proinflammatory cytokines (e.g. interleukin-6 (IL-6) and tumor necrosis factor-α (TNF-α)). SARS-CoV-2 enters into human cells and causes an activation of the cells of innate and adaptive immunity. Macrophages and dendritic cells that belong to the first group, through the Toll-like receptor (TLR) 3, 7 and 8, recognize the viral RNA. This binding results in the activation of the inflammasome and the production of cytokines such as IL-1β, IL-6 and TNF-α. The production of these molecules is necessary for the human body to protect itself from the viral invasion not only at the respiratory level but also in other organs affected with SARS-CoV-2 such as the heart, blood vessels, kidneys and brain [32,33].

During severe COVID-19 a hyperactivation of the immune system has been observed, together with an excessive production of cytokines, which causes negative effects on the body such as multi-organ failure and ARDS [33].

Older adults tend to develop severe forms of COVID-19 and have stronger hyper inflammation (the so-called ‘cytokine storm’) and hymmune response as compared to young people. In these subjects, there is a more suitable environment for the activation of the inflammosome than in younger adults, through the TLR receptors and through the viroporins, which are ion channels built by the virus in host cells and that release K + into the extracellular fluid, changing the ionic balance and activating the inflammosome. In the older adults some conditions such as aging, renal disease, reactive oxygen species (ROS) unbalance and hyperproduction, facilitate this electrolyte imbalance and the cytokines hyperproduction [34].

A reduced immune response is instead a peculiar characteristic of very old patients; they commonly present less ground glass opacities than younger counterparts on CT findings [35].

### Microbiota and gut dysbiosis in older adults

Intestinal microbiota alterations related to aging can be associated with chronic systemic inflammation, oxidative stress, insulin resistance and may negatively impact on muscle protein synthesis and function [36–38].

It has been hypothesized that gut dysbiosis is also associated with altered inflammation in older adults with COVID-19 and contributes to the alteration of the immune system and the severity of the disease. Recent evidence has revealed that microbiota changes are linked to superinfections in COVID-19 patients, especially in older adults. Several studies suggest that SARS-CoV-2 infection could cause a decline in gut microbiota diversity leading to an increase of opportunistic bacteria such as Actinomyces Streptococcus, Rothia, Enterococcus and a decrease of some species such as Faecalibacterium, Eubacterium, Coprococcus and Ruminococcus [39]. This dysregulation leads to a high risk of blood superinfections with a migration of bacteria into the bloodstream [40]. In older COVID-19 patients there is also a depletion of butyrate-producing bacterias [41], which are not able to produce short-chain fatty acids (SCFAs), responsible for promoting the integrity of the intestinal barrier through the differentiation of intestinal epithelial cells. Therefore older adults could have a more significant intestinal barrier impairment than younger people and SARS-CoV-2 infection enhances this unbalance by invading the enterocytes, promoting gut damage and dysbiosis [42]. Also comorbidities which are most commonly observed in older adults such as diabetes, obesity and kidney disease, can promote gut dysbiosis and intestinal dysfunction [43]. The complex interplay between COVID-19, gut dysbiosis and aging can pass also through the respiratory system [44] ACE2 receptors are diffused ubiquitously but are predominant at respiratory level. Intestinal microorganisms could regulate systemic and pulmonary inflammatory responses, and respiratory infections can be associated with altered gut microbiota in a vicious cycle. The complex dialogue between gut and lungs could be defined as a ‘gut-lung axis’ and the loss of homeostasis which can occur especially in older adults could be associated with significant dysregulation of immunity, host cell invasion and damage from SARS-CoV-2, and occurrence of fatal opportunistic infections [45–47].

**Figure 1.**
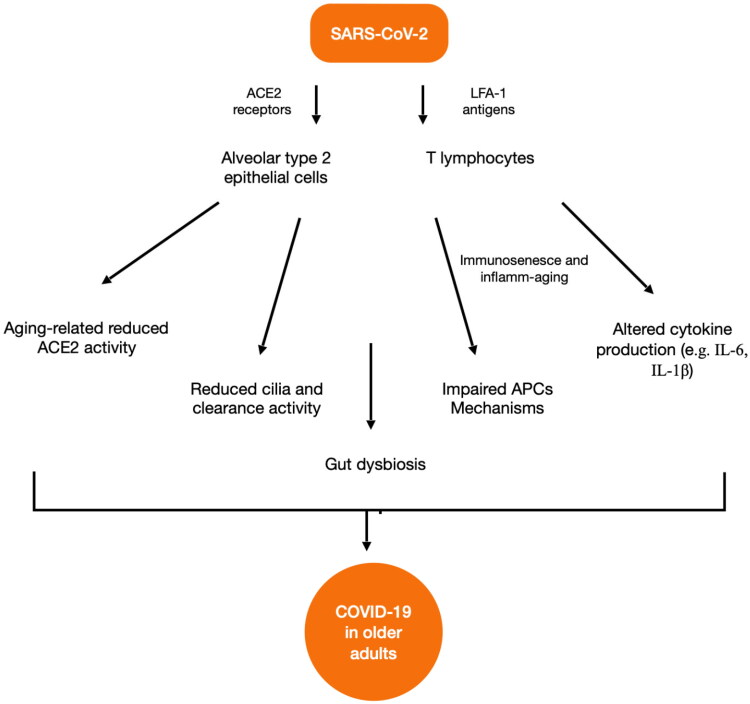
Aging-related mechanisms of disease from SARS-CoV-2. SARS-CoV-2: severe acute respiratory syndrome coronavirus 2; ACE2: angiotensin-converting enzyme 2; LFA-1: lymphocyte function-associated antigen 1; APC: antigen presenting cells; IL: interleukin; COVID-19: coronavirus disease 2019.

There is evidence also from autopsy studies that gut dysbiosis related to corticosteroid therapy, persistent mechanical ventilation, prolonged hospital stays and lymphocyte depletion is associated with fatal COVID-19 related to opportunistic infections [48,49]. Figure 1 shows the main aging-related mechanisms which may be involved in promoting severe forms of COVID-19 in older adults.

## COVID-19 vaccination in older adults

Vaccination campaigns against SARS-CoV-2 have significantly reduced globally the number of deaths and the risk of severe disease, but there is still a great field of uncertainty in the older age, especially in frailty subjects. Only a limited percentage of frail older patients has been included in the vaccine arms of clinical trials [50], specifically 5% of patients aged ≥ 70 and 24,8% aged ≥ 65 years in the Oxford-Astrazeneca and Moderna trials, respectively [51,52].

The real-word evidence documents that some older adults are still hospitalized for severe COVID-19 and have a significant risk of death despite full vaccination [53].

Tang et al. have evaluated the SARS-CoV-2 vaccine effectiveness during the Delta wave according to frailty status in a total of 58,604 U.S. veterans (mean age: 58.9 ± 17.0, 87.5% men). Among these, 27.8% patients were in prefrail, and 24.9% in frail conditions. The authors found a reduced mRNA vaccine effectiveness against the Delta variant in frail and pre frail patients (respectively 62.8% (95%CI: 59.8–65.7), and 73.9% (95%CI: 72.0–75.7)). The effectiveness was highest in robust patients, reaching 77.0% (95%CI: 75.7–78.3) [54].

The mechanisms underlying the lack of a complete vaccine effectiveness in some group of patients are still unknown. Immunosenescence and inflamm-aging can be linked to an altered immune response against the vaccination. Adaptative immune system decreases with age and data indicate that antibody responses decline after a booster dose in adults older than 60 years [55,56].

Another issue to consider when administering vaccines to older adults is the susceptibility to adverse events (AEs). A previous study enrolling 134 frail patients older than 65 years (mean age 82.9 years, 71.6% female) and residents in Spanish long‐term care facilities (LTCFs), excluded a significant increase of adverse reactions in this group. Factors such as older age, frailty, cognitive impairment and comorbidities were not associated with significant changes in antibody titers, and the authors concluded that the BNT162b2 mRNA COVID‐19 vaccine was effective and safe in older adults with frailty. However, these data were taken with caution given the limited sample size of the study [57].

More recently, a large phase 2, randomized clinical trial has documented a significant higher rate of AEs in at-risk patients with at least one Charlson Comorbidity Index condition [58]. Furthermore, recent European and American pharmacovigilance systems have revealed a significant higher risk of post-vaccination hospitalizations, life-threatening outcomes, and deaths among patients aged over 65 years than younger people, and AEs onset occurred usually within the first 7 days from vaccination in 77.6–89.1% of cases [59].

## Evaluation of frailty in patients with COVID-19

A Comprehensive Geriatric Assessment (CGA) is a multidimensional (multidomain) approach aimed at evaluating specifically the different patient’s domains such as functional ability, physical, cognitive and mental health, and also other domains such as financial status, social support and environmental factors [60].There is not a gold standard method to assess frailty, but there are two principal models: Fried’s phenotype (FP) and the cumulative deficit model or frailty index (FI). In the former, frailty is based on five components: weight loss, exhaustion, poor hand-grip, low physical activity and slow walking speed. The latter includes symptoms, signs, disability and comorbidities [60].

Some methods are especially useful to identify frailty among patients with COVID-19, the most important are the Clinical Frailty Scale (CFS), which gives a score to patients on the basis of their daily functions and is a useful tool to identify subjects at risk of mortality for COVID-19, the Frail Scale (FRAIL), which is a simple and low-cost method including five criteria that categorize a patient in robust, pre-frail and frail, and the Edmonton Frailty Scale (EFS), which reveals the risk of frailty by investigating nutrition, mood and drugs [60].

The most common instrument to measure frailty in COVID-19 patients and to predict their prognosis is CFS [60]. Several studies have reported an association between frailty measured with CFS and risk of hospitalization and mortality in older subjects. A perspective study which included randomly 255 COVID-19 patients (mean age 66 years ± 17 years) who were hospitalized and followed-up for 60 days showed that 90% of all deaths occurred in patients with an age over 65 years, and that CFS was the strongest predictor element for death at multivariate analyses [61].

In another single-center prospective observational cohort study which enrolled 729 patients aged ≥80 years, 251 were frail according to the CFS. The evaluation of frailty in the Emergency Department (ED) was useful to predict the risk of in-hospital death in older COVID-19 adults [62]

In a retrospective cohort study of 103,561 COVID-19 patients (mean age 84.1, 50% female) hospitalized in England, the authors examined the relationship between frailty and COVID-19 mortality using the Hospital Frailty Risk Score (HFRS), a method which records ICD-10 codes for dividing patients into three frailty risk categories (low (< 5), intermediate (5–15), and high (> 15)). Intermediate and high frailty risk categories had reduced survival days compared to those without frailty (accelerated failure time estimates for intermediate and high risk categories of 0.63 (95% CI 0.58–0.68) and 0.67 (95% CI 0.62–0.72), respectively) [63].

The risk of death driven by frailty seems to be enhanced by the presence of comorbidities [64–66]. The retrospective, cohort COVIDAge study, which enrolled 235 Caucasian patients (mean age of 86 ± 6.5 years, 43% male) has found an increased rate of death (32%) in those patients who were frail at baseline and had a higher comorbidity burden and worse functional status (all *p* < 0.001). Also delirium was a predictor of death (*p* = 0.007) and a higher prevalence was observed of heart failure and peripheral artery disease (*p* = 0.044 and *p* = 0.009, respectively) [67].

In the GeroCOVID study, disease severity was not associated with loss of autonomy in survived COVID-19 older patients; on the contrary, the pre-morbid ADL and frailty had a primary role in the loss of ADLs [68].

## The treatment of COVID-19 in older adults

Since the pandemic outbreak, researchers have tried to identify strategies able to reduce the risk of clinical deterioration from COVID-19, hospitalization and mortality. Before the development of an effective campaign of vaccination, some drugs already available on the market were urgently evaluated and employed in order to try to improve the outcome of COVID-19 patients, especially older adults [69].

The use of hydroxychloroquine, an antimalarial drug, was suspended as it did not show an improvement in survival, and was associated with important AEs such as prolonged QT interval and the risk of secondary arrhythmias [69].

Other pharmacological classes were considered, including antivirals, anticoagulants and anti-inflammatory drugs.

### Corticosteroids

The use of dexamethasone remains the mainstay of treatment in hospitalized patients having a severe COVID-19 pneumonia. Very old patients, despite full vaccination and/or not having taken antiviral drugs in an early phase of the disease (see below), can still have a severe lung disease with respiratory failure, needing oxygen and/or ventilation and may benefit of cortisone therapy in a late (hyper inflammation) stage of the disease. The RECOVERY trial has demonstrated efficacy in reducing the mortality at 28-day of dexamethasone therapy in those who need either invasive mechanical ventilation or oxygen. Among 2104 patients of the RECOVERY trial who were assigned to dexamethasone, 469 and 494 were aged between 70 and 79 years and ≥ 80 years respectively (22 and 23%). Age-stratified data were not available, therefore any additional benefit in older adults was not reported [70]. A recent observational study has found a mortality risk reduction in patients with > 7 days of symptom onset to initiation of corticosteroids (HR 0.56, 95% CI 0.33–0.95; *p* = 0.03). especially in mechanically ventilated patients (HR 0.38, 95% CI 0.24–0.60; *p* < 0.001), therefore a time > 7 days from symptom onset should suggest a cortisone treatment [71]. An early cortisone treatment was not associated instead with significant differences in terms of in-hospital mortality and intensive care unit admission with controls (27.1% vs. 22.8%, respectively, *p* = 0.63) [72]. Moreover, cortisone treatment may have deleterious and catabolic effects especially in older adults, increase the risk of bacterial infections, be associated with hyperglycemia and affect the risk-benefit ratio, especially in frail subjects. Maintaining a good hydration, monitoring kidney function and deprescribing drugs that may contribute to a worsening of a coexistent renal disease are useful measures which can increase the drug tolerability and therapeutic efficacy [71].

### Antiviral drugs

The first antiviral drug which was approved for COVID-19 in patients at high risk of progression to severe disease was remdesivir, a viral RNA polymerase inhibitor and adenosine nucleotide analogue [73]. Two different therapeutic schemes have been adopted according to the treatment setting, in versus outpatient, both without the need for oxygen therapy but with a positive nasopharyngeal swab for SARS-CoV- 2 and high risk of severe disease progression. In the first setting, the treatment is carried out for a total of 5 days with a first 200 mg intravenously dose followed by 100 mg per day for 4 days. In the outpatient setting, the drug is administered orally for 3 days. In a study, the use of remdesivir was associated with a significant clinical improvement at 28 days in patients without and those with low-flow oxygen, and patients with low-flow oxygen therapy had a significant reduction of mortality risk compared to controls (aHR 0.85, 95% CI, .77–.92; 28-day mortality 8.4% [865 deaths] vs 2.5% [1334 deaths] for remdesivir patients and for controls, respectively) [73].

The effectiveness and safety of this drug in older adults are still largely unknown, even though some studies have shown efficacy in reducing the risk of severe COVID-19 disease in this population. The SEMI-COVID-19 Registry, a large retrospective analysis of 4331 SARS-CoV-2 vaccine-free patients (1312 aged ≥ 80 years), who were hospitalized for COVID-19 between July and December 2020 in Spain, has shown that 140 very old patients who were treated with remdesivir had a lower mortality risk than those were not treated (OR (95% CI): 0.45 (0.29–0.69)) [74].

Against beneficial effects, remdesivir can cause severe hepatic and/or renal dysfunction, and the drug should be used with caution especially in the first days of treatment, with monitoring of liver and kidney function. Interestingly, there is low data about the safety profile of remdesivir in very old patients. A study enrolling 80 patients of whom only 32.5% were aged ≥ 80 years has shown that liver dysfunction induced by remdesivir occurred in 36.3% of all-age groups, and that there were no significant differences in terms of AEs between younger and older patients [75].

In nonhospitalized patients, other two drugs have been approved in COVID-19 patients at risk of progression to severe disease: Paxlovid (Nirmatrelvir/Ritonavir) and Molnupiravir (Lagevrio). Both drugs have been indicated in patients who do not require supplemental oxygen therapy and should be administered within 5 days of the onset of symptoms. Despite the first positive results of the MOVe-OUT study [76], however, and based on the totality of data, Molnupiravir (Lagevrio) has been recently suspended by the Committee for Medicinal Products for Human Use of the European regulatory body (CHMP) and from the European Medicines Agency (EMA) due to the lack of benefits in terms of reduction of hospitalization risk, duration of illness or mortality in adults at risk of severe COVID-19 [77].

Paxlovid (Nirmatrelvir/Ritonavir) has resulted instead in a risk reduction of 89% versus placebo of progression to severe Covid-19, without significant safety concerns [78].

The recommended dose is 300 mg of Nirmatrelvir (2 × 150 mg tablets) with 100 mg of ritonavir (1 × 100 mg tablet) taken together orally every 12 h for a total of 5 days.

Nirmatrelvir inhibits the 3CL main protease (Mpro) from SARS-CoV-2 and reduces the ability of SARS-CoV-2 to replicate in human cells, while ritonavir is a strong inhibitor of cytochrome P450 (CYP) 3A4 and acts as a strong pharmacokinetic boosting agent, having already been used to enhance HIV protease inhibitors [79].

The drug was effective to reduce hospitalization rates and risk of death associated with COVID-19 in patients aged ≥ 65 years during the omicron surge (14.7 versus 58.9 cases per 100,000 person-days among treated and non treated patients, respectively (adjusted HR, 0.27; 95% confidence interval [CI], 0.15–0.49), but gave no benefits in younger adults [80]. The use of this drug is limited by the interactions with other drugs, an issue of particular interest in older adults where comorbidities are often associated with polypharmacy, and an anticipatory deprescribing of interacting drugs could be useful to optimize the effectiveness of nirmatrelvir [81,82].

### The use of monoclonal antibodies (MonoAbs)

Another early therapeutic option which can be used early to reduce severe forms of COVID-19 in hospitalized patients is represented by monoclonal antibodies (MonoAbs), which should be given within 7 days of the onset of symptoms. The administration after 7 days can be recommended only in patients with negative serology for SARS-CoV-2 and prolonged molecular swab positivity, and in subjects with immunodeficiency [83]. Some therapeutic options are casirivimab/imdevimab (effective vs the Delta B.1.617.2, Alpha B.1.1.7., Gamma P.1 and Beta B.1.351 variants), bamlanivimab/etesevimab (effective vs Delta B.1.617.2 and Alpha B.1.1.7.) and sotrovimab (effective vs variants Omicron B.1.1.529, Delta B.1.617.2, Alpha B.1.1.7., Gamma P.1 and Beta B.1.351). The administration is unique for all drug combinations, with three different dosages: bamlanivimab 700 mg + etesevimab 1400 mg intravenously; casirivimab 600 mg + imdevimab 600 mg intravenously or subcutaneously and sotrovimab 500 mg intravenously.

The use of MonoAbs represents a valid therapeutic option in older adults [84]. With particular focus on very old adults, a single-center retrospective observational study conducted in France among 36 older adults (mean age of 82.6 ± 9.5 years with 80% of patients ≥75 years) who received sotrovimab from January to March 2022, has shown that none of the hospitalized patients were admitted to the intensive care unit. In addition, there were no significant AEs after drug administration [85].

The PROVENT trial, a large, randomized phase 3 study evaluating one dose of Tixagevimab-Cilgavimab (Evusheld) vs placebo in 5197 participants with an increased risk of an inadequate SARS-CoV-2 vaccination response and/or an increased risk of exposure, has found a significant risk reduction of symptomatic COVID-19 cases in drug vs placebo-treated patients (relative risk -RR- reduction, 76.7%; [95% CI, 46.0–90.0]; *p* < 0.001). The effect was highest at six months of follow-up (RR reduction of 82.8% [95% CI, 65.8–91.4]). Median participant age was 53.5 ± 15.0 years, and adults aged ≥ 60 years had a higher RR reduction than those aged < 60 (87.8 [95% CI 56.9–96.6], vs 79.6 [95% CI 53.5–91.1] years, respectively) [86].

Furthermore, the recent Efficacy and safety of intramuscular administration of tixagevimab-cilgavimab for early outpatient treatment of COVID-19 (TACKLE) study has found a significant reduction of progression to severe disease or death in drug-treated (4% out of 407 participants) versus placebo-treated (9% out of 415 participants), both unvaccinated and SARS-CoV-2 positive patients (RR reduction of 50.5% [95% CI 14·6–71·3]; *p* = 0.0096, absolute risk reduction of 4·5% (95% [CI 1·1–8·0]; *p* < 0.0001) [87].

EMA therefore recommends the use of EVUSHELD both for pre-exposure prophylaxis of COVID-19 in subjects with a high risk of inadequate response to active immunization (e.g. subjects aged ≥ 60 years with comorbidities, preexisting chronic disease, immunocompromised, or with vaccination intolerance) and for the treatment of COVID-19 adults and adolescents aged ≥ 12 years with a weight of at least 40 kg who are at risk of severe COVID-19 but do not require oxygen therapy [88].

### Non pharmacological measures

Paucisymptomatic or asymptomatic older adults with COVID-19 at home should be carefully evaluated. Good hydration, avoiding malnutrition and trying to keep stable the underlying disorders are useful measures to reduce the risk of hospitalization due to worsening of the clinical picture [89], and to keep a good functional status in older adults in general [90]. Frail patients who are hospitalized for COVID-19 are at higher risk of hyperactive delirium, which can be associated with risk factors such as acute urinary infection, hospitalization itself, lung failure and hypoxia, dehydration and pain [91]. The establishment of measures aimed at reducing these risk factors is suggested for reducing the risk of delirium, which is associated with increased in-hospital mortality risk from COVID-19 in older adults especially if combined with physical frailty [91].

A thorough clinical evaluation and a caregiver phone call can promptly identify a worsening of the clinical condition [90]. Peripheral saturation (SO2) measurement and scales such as the 6-min walking test and the Modified Early Warning Score (MEWS) remain good easy-to-use instruments to evaluate the risk of deterioration from COVID-19. However peripheral saturation is not always a reliable parameter especially in older adults having comorbid conditions such as heart disease or chronic pneumonia which could be associated with hypoperfusion, hypothermia and tremor [92] A bedside ultrasound assessment can be useful to assess COVID-19 patients [93]. The lung ultrasound (LUS) score has been shown to effectively identify those patients having the highest risk of mortality (LUS score ≥20), but requires availability of US devices at home and an adequate training [94–96].

## Conclusion

The SARS-CoV-2 vaccination has radically reduced the clinical impact of the current pandemic and has improved the prognosis in patients who are still affected with the disease. However, the issue remains still open in older patients with frailty and/or comorbidities and very old patients, whocan be still hospitalized with severe COVID-19 despite full vaccination. Current treatment strategies include the early use of monoclonal antibodies, which are effective and safe in patients, especially nonvaccinated, and older adults. The inclusion of older patients in specific trials aimed at evaluating the impact of COVID-19 in vulnerable categories is recommended, and clinicians should be encouraged to establish personalized care plans based on a comprehensive geriatric assessment, tailored interventions and specific therapeutic algorithms among older adults.

## Data Availability

All figures and tables are original and are not taken from other publications. Data sharing is not applicable to this article, as no new data were created or analysed in this study.
